# New Insights into the Susceptibility of Immunocompetent Mice to Usutu Virus

**DOI:** 10.3390/v12020189

**Published:** 2020-02-08

**Authors:** Emna Benzarti, Michaël Sarlet, Mathieu Franssen, Daniel Desmecht, Jonas Schmidt-Chanasit, Mutien-Marie Garigliany

**Affiliations:** 1 Fundamental and Applied Research for Animals & Health (FARAH), Faculty of Veterinary Medicine, University of Liège, Sart Tilman B43, B-4000 Liège, Belgium; benzarti_e@yahoo.com (E.B.); Michael.Sarlet@uliege.be (M.S.); mfranssen@uliege.be (M.F.); daniel.desmecht@uliege.be (D.D.); 2 Bernhard Nocht Institute for Tropical Medicine, WHO Collaborating Centre for Arbovirus and Haemorrhagic Fever Reference and Research, 20359 Hamburg, Germany; jonassi@gmx.de; 3 Faculty of Mathematics, Informatics and Natural Sciences, University of Hamburg, 20354 Hamburg, Germany

**Keywords:** Usutu virus, immunocompetent, mice, infection, encephalitis

## Abstract

Usutu virus (USUV) is a mosquito-borne flavivirus that shares many similarities with the closely related West Nile virus (WNV) in terms of ecology and clinical manifestations. Initially distributed in Africa, USUV emerged in Italy in 1996 and managed to co-circulate with WNV in many European countries in a similar mosquito–bird life cycle. The rapid geographic spread of USUV, the seasonal mass mortalities it causes in the European avifauna, and the increasing number of infections with neurological disease both in healthy and immunocompromised humans has stimulated interest in infection studies to delineate USUV pathogenesis. Here, we assessed the pathogenicity of two USUV isolates from a recent Belgian outbreak in immunocompetent mice. The intradermal injection of USUV gave rise to disorientation and paraplegia and was associated with neuronal death in the brain and spinal cord in a single mouse. Intranasal inoculation of USUV could also establish the infection; viral RNA was detected in the brain 15 days post-infection. Overall, this pilot study probes the suitability of this murine model for the study of USUV neuroinvasiveness and the possibility of direct transmission in mammals.

## 1. Introduction

Usutu virus (USUV) is a mosquito-borne flavivirus of the *Flaviviridae* family and is closely related to WNV [1]. Similar to WNV, its enzootic cycle involves wild birds as reservoirs and a wide range of mammals as accidental hosts [2,3,4,5,6,7], including humans [8]. Since its discovery in 1959, it has been isolated from mosquitoes and birds in Europe [9,10], Africa [11], and the Middle East [12]. Until now, USUV has never been detected in the United States, but the events of its introduction, endemization, and co-circulation with related flaviviruses, such as the St. Louis encephalitis virus and WNV, might occur in the future [13].

USUV appears to be pathogenic and lethal to certain wild bird species [14,15] while it often causes asymptomatic infections in humans [16]. Nevertheless, a few cases of neurological disease in both immunocompetent and immunocompromised human patients have been reported [17,18]. It is worth mentioning that none of the recent outbreaks of other arboviruses, such as the Zika virus and WNV, were predicted [19]. Thus, the evidence of USUV zoonotic potential and pathogenicity in birds warrants investigations on its transmission, neuropathogenesis, and countermeasures using study models to reduce the economic and sanitary burden it may pose in the future.

Experimental infections have shown that USUV pathogenicity is rather limited in immunocompetent mammals. Fruit-eating African bats could not be experimentally infected with USUV [20]. Similarly, wild-type mice showed nil or limited susceptibility when challenged with low or high doses of USUV via the intraperitoneal route (i.p.) [2,21,22,23,24,25], including USUV prototype strain SAAR-1776 (*GenBank:* AY453412) [21,22,24], which was isolated by intracerebral inoculation of newborn mice [22]. However, in the study of Diagne et al. [2], both subcutaneous and i.p. infections using 10^3^ PFU of this strain resulted, respectively, in 30% and 50% of mortalities in 3–4-week-old Swiss Webster (CFW) mice after 15 days of infection [2]. Similarly, in the same study, the i.p. inoculation of USUV strain HB81B8 (*GenBank:* KC754955) induced 10% of mortality 10 days after the infection [2]. These findings evince that the outcome of USUV infection in immunocompetent mice can be highly dependent not only on the viral strain or dose but also on the mouse line and age. As a consequence, while no signs nor mortality were observed after the i.p. challenge of wild-type 6-week-old 129/Sv mice with 10^4^ PFU of the USUV strain Biotec (*GenBank:* KU760915) [23], the susceptibility of this model to other representative USUV strains currently circulating in Europe still remains to be investigated.

The intracerebral route was successfully used to induce signs and mortalities due to USUV infection [2,22]. This route could not, however, mimic the naturally occurring disease in humans as this inoculation only reflects viral neurovirulence, whereas the outcome of peripheral inoculation (e.g., subcutaneous or i.p.) reflects both neurovirulence and neuroinvasiveness [26]. Thus, researchers have capitalized on the ability of suckling mice [21,25] or mice lacking the interferon α/β receptor (IFNAR-/-) [23,27] to model USUV neuroinvasiveness and neuropathogenicity [25] and to test the effect of some antiviral [27] and vaccine [23] candidates. However, the lack of a fully functional immune response in these animals hinders their ability to accurately model disease pathogenesis and to investigate the efficacy of certain vaccine candidates [28].

Cutaneous infection by the intradermal (i.d.) injection presumably better mimics natural infection in humans with mosquito-borne pathogens, including WNV [29,30]. The intranasal inoculation (i.n.) has been utilized to evaluate the potential for aerosol transmission of numerous arboviruses [31]. These two routes have not yet been utilized to infect mice with USUV.

In this report, we describe the pathological phenotype of two phylogenetically distinct strains of USUV in immunocompetent mice using either i.p., i.d., or i.n. routes of inoculation. 

## 2. Materials and Methods 

### 2.1. Viruses

USUV strains USU-BE-Seraing/2017 (*GenBank:* MK230892, Lineage: Europe 3) and USU-BE-Grivegnee/2017 (*GenBank:* MK230891, Lineage: Africa 3) isolated from two European blackbirds (*Turdus merula*) during an avian outbreak in 2017 in Belgium were used for the challenge of mice [4]. The viruses were amplified in African Green Monkey Vero cells (ATCC^®^ CRL-1586; passage number 3), titrated by the 50% tissue culture infective dose (TCID_50_) technique and stored at −80°C. 

### 2.2. Mouse Experiments

Wild-type 129/Sv mice, purchased from Charles River Laboratories (France), were kept in the biosafety level 2 (BSL-2) experimental animal facility of the Department of Pathology, Faculty of Veterinary Medicine, Liège, Belgium. Isoflurane inhalation was used for anesthesia prior to the infections. Six groups of 6 female 4–5-week-old mice were inoculated with 10^6^ TCID_50_ of USUV (strain “Seraing” or “Grivegnee”) via the i.p., i.d. (in the lower back) or i.n. routes. The inoculums were dispersed in 100 µL of cell culture medium (Dulbecco’s Minimum Essential Medium (DMEM) supplemented with 1% penicillin/streptomycin). To ensure proper intradermal injection, each inoculum was injected into two separate sites, with approximately 50 µL in each site. Three different control groups of 6 age-matched female mice were injected with an equivalent volume of medium without a virus via the i.p., i.d., or i.n. routes. During the experiments, all animals were monitored daily, weighed, and received water and food ad libitum. Fresh urine and feces samples were collected daily for virus detection. Any mouse showing more than 20% of weight loss was anesthetized then euthanized, as were all surviving animals 15 days after the infection. Mice were bled prior to infection and euthanasia for serological and/or real-time reverse transcription-polymerase chain reaction (RT-qPCR) analysis. Brain, spinal cord, lung, heart, liver, spleen, kidney, and small intestine samples were collected from the infected animals and processed for histological and immunohistochemical analysis. Portions from the brain were also frozen at −80°C for RT-qPCR assay. Mock-inoculated mice were euthanized at the end of the experiment and blood, liver, and brain samples were taken for RT-qPCR analysis. The animal care and experiments were approved and supervised by the Committee for Ethics in Animal Experimentation of the University of Liege, Belgium (Identification code: 18-2018, permission date: 31/10/2018).

### 2.3. Histopathology and Immunohistochemistry

Tissue samples were fixed in 10% neutral buffered formalin, embedded in paraffin wax, sectioned, and then stained with hematoxylin and eosin. For antigen detection, slides were processed for immunohistochemistry (IHC) as described in [32].

### 2.4. Viral Detection by RT-qPCR and Isolation in Vero Cells

For USUV genome detection, total RNA was extracted from serum, urine (200 µL), tissue, and feces (50 mg) samples and the viral genome load was absolute-quantified by RT-qPCR using a standard curve, which was constructed as described in [33]. Virus isolation on Vero cells [4] was attempted using urine and feces samples.

### 2.5. Antibodies Detection

Serum samples collected prior to the infection or at the end of the experiment were screened for antibodies to USUV using a competitive ELISA kit (ID Screen^®^ West Nile Competition Multi-species, Grabels, France) following the manufacturer’s instructions. The plates of this kit are pre-coated with the WNV envelope protein, which cross-reacts with immunoglobulins M and G against viruses from the Japanese Encephalitis Viruses serocomplex, including USUV [34,35].

### 2.6. Statistical Analysis

Statistical analysis was performed using the Shapiro–Wilk test for normality followed by the non-parametric Kruskal–Wallis test and paired t-tests (post hoc comparisons) implemented in *r studio* to define differences between viral RNA copies in the brain from 3 independent groups of subjects. Significance was defined by *p* < 0.05.

## 3. Results

### 3.1. Mortality Rates

One mouse infected with USUV strain USU-BE-Seraing/2017 via the i.d. route showed a weight drop (from 14.64 to 13.28 g), disorientation and half-closed left eye at day 6 post-infection. By day 8, this mouse showed paresis of the posterior body and loss of 20% of the initial body weight and was euthanized and autopsied. The remaining mice had no clinical signs and gained weight during the experiment (data not shown). The control group remained alive and asymptomatic until the end of the experiment.

### 3.2. Pathological Findings and Antigen Detection by IHC

**Figure 1:**
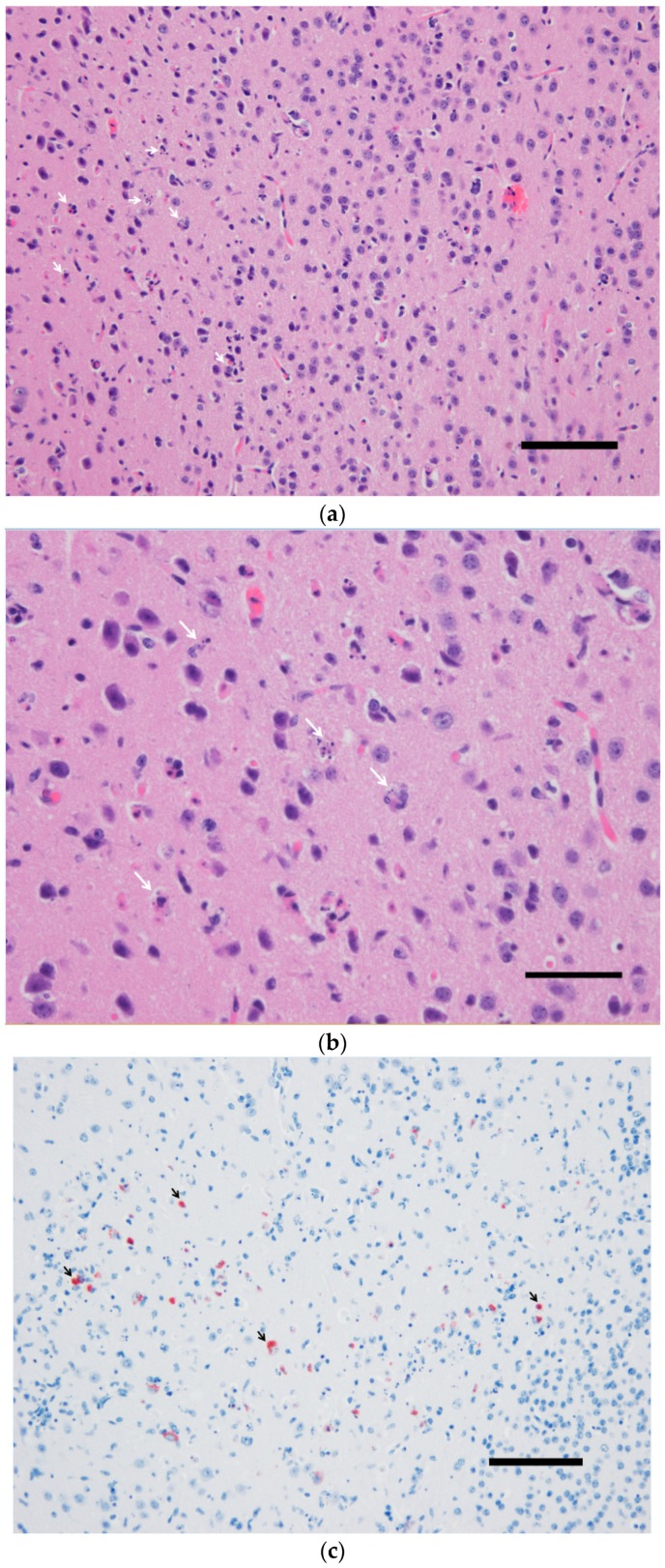

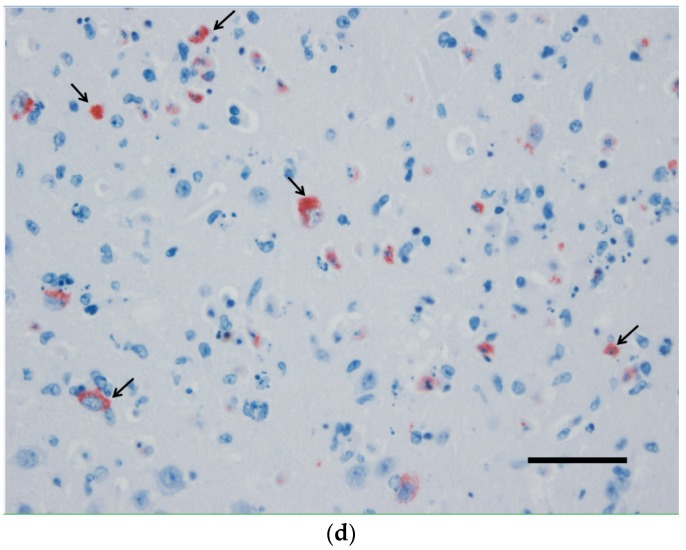
The brain of a wild-type 129/Sv mouse injected with the Usutu virus via the intradermal route. Massive neuronal death demonstrated by karyorrhexis and karyolysis (**a**,**b**) in correlation with intense immunohistochemical labeling of USUV antigens (**c**,**d**). (**a**,**b**) Hematoxylin and eosin staining, (**b**,**c**) hematoxylin counterstain. Scale bars a and c = 200 µm, magnification 100×; Scale bars b and d = 50 µm; magnification 200×.

**Figure 2:**
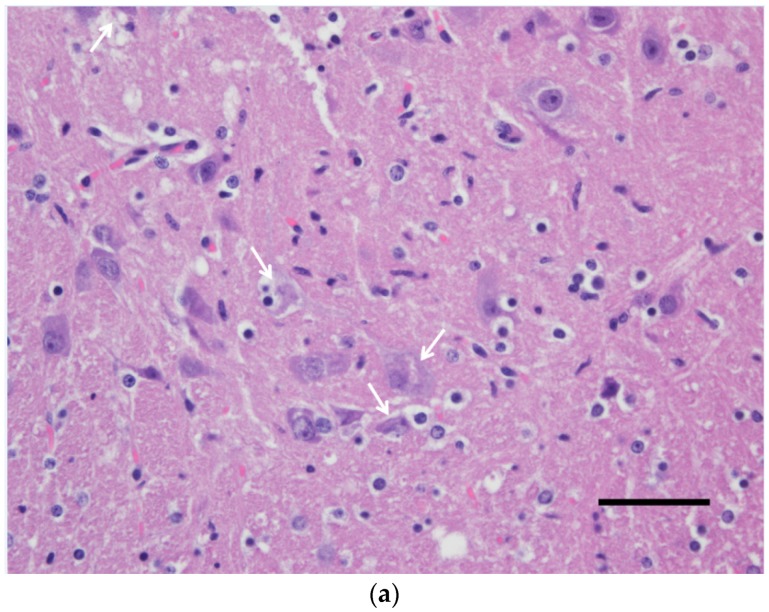

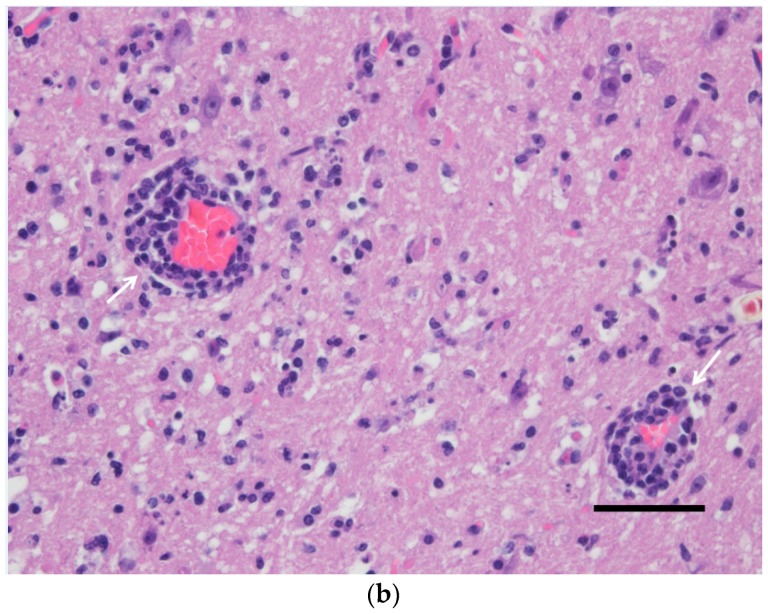
Spinal cord (gray matter) of a wild-type 129/Sv mouse injected with the Usutu virus via the intradermal route. Abundant neuronal death with neuronophagia and moderate satellitosis and gliosis (**a**) and lymphoplasmacytic perivascular cuffs (**b**). Hematoxylin and eosin staining. Scale bars = 50 µm, magnification 200×.

While no gross lesions could be observed upon the necropsy of the sick mouse, extensive neuronal death and strong USUV antigen signals were observed in the brain (Figure 1). Similar pathological findings in the spinal cord of this specimen were found (Figure 2) but only a few neurons were successfully stained using IHC (not shown).

The remaining mice showed no gross or microscopic lesions on day 15 post-infection. Immunohistochemical staining of USUV antigens in their tissues was negative as well.

### 3.3. Viral Detection by RT-qPCR and Isolation in Vero Cells

The specimen euthanized on day 8 post-infection presented high RNA loads detected by RT-qPCR in the brain (9.38 ± 0.09 log10 VRC/50 mg), liver (4.15 ± 0.11 log10 VRC/50 mg), lung (4.47 ± 0.08 log10 VRC/50mg), spleen (4.49 ± 0.07 log10 VRC/20 mg), kidney (6.36 ± 0.13 log10 VRC/50 mg), intestine (5.1 ± 0.17 log10 VRC/50 mg), and blood (4.99 ± 0.10 log10 VRC/mL). No infectious virus could be isolated from the urine, feces, and serum using Vero cell cultures.

**Figure 3:**
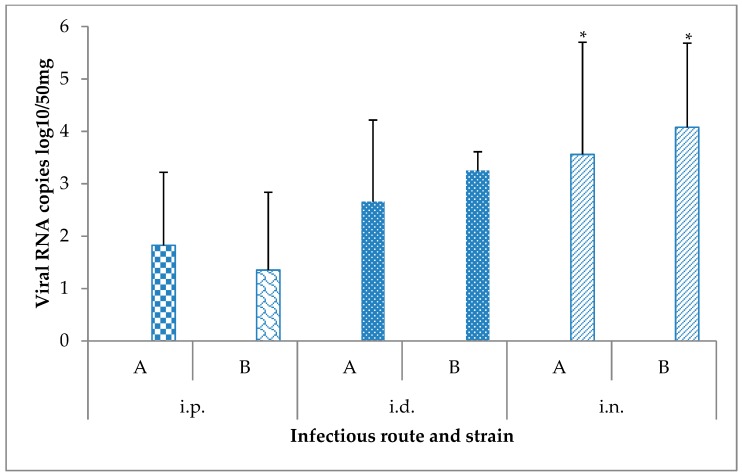
Viral RNA loads measured by RT-qPCR in brain samples (*n* = 6 per condition) collected from mice 15 days after their challenge with the Usutu virus via different routes. * *p*-value < 0.05. A: USU-BE-Seraing/2017, B: USU-BE-Grivegnee/2017, i.d.: intradermal, i.n.: intranasal, i.p.: intraperitoneal.

No evidence of virus circulation was found by means of RT-qPCR in the blood of mice euthanized at 15 days following their infection. Similarly, blood, liver, and brain samples from the mock-inoculated groups euthanized at the end of the experiment were USUV-negative using the RT-qPCR. By contrast, the USUV genome was detected in the brains of the infected mice (Figure 3).

While comparable RNA loads were found in the brain of mice infected with both USUV strains (*p* = 0.25), significant differences in RNA copy numbers in this organ were detected depending on the infection route (*p* = 0.0018). The i.n. route resulted in higher RNA loads in the brain compared to the i.p. and i.d. routes (*p* = 0.0092 and *p* = 0.03, respectively). In addition, significantly higher viral RNA copies were detected in this tissue with the i.d route when compared to the i.p. route (*p* = 0.035).

### 3.4. Antibody Detection

All mice were negative for antibodies against USUV at the beginning of the experiment. The number of seroconverting specimens after 15 days of the infection was variable according to the injection route (Table 1).

## 4. Discussion

The limited virulence of both USUV strains used in this experiment to adult wild-type 129/Sv mice is in accordance with other studies using NMRI mice aged 2 weeks or more [25] and adult Swiss mice (5–6 [22], 8 [21] or 10 [24] weeks old). One of the reasons for the resistance of immunocompetent mice is the IFN response that plays a major role in the control of the in vivo pathogenesis of USUV, as well as other flaviviruses such as Zika virus [28]. In fact, contrary to immunocompetent mice, high mortality rates were observed after USUV infection in suckling mice (which have not yet developed a functional IFN response [21,25]), or in mice knocked-out for the IFN-α/β and/or IFN-γ pathways [23,27]. Nonetheless, our study could illustrate the neuroinvasiveness and neurovirulence of USUV in an immunocompetent mouse injected via the i.d. route. In naturally infected birds, systemic infection with neuronal necrosis and encephalitis are often observed [4,36]. Here, lesions were seen in the central nervous system (CNS), while histopathology and IHC revealed no peripheral viral replication, indicating a selective infection in the CNS, in a similar manner as described in suckling mice experimentally infected with USUV [25]. However, high RNA loads were detected by RT-qPCR in the liver, lung, and spleen. These RNA loads might at least in part be associated with the RNAemia and residual blood in these tissues, although mice were bled prior to euthanasia. Further, despite RNA detection in the kidney, intestine, and blood, no infectious virus could be isolated from the urine, feces, and serum using Vero cell cultures. These findings can be explained by the higher sensitivity of RT-qPCR over cell culture and IHC assays or might reflect the presence of viral RNA without viral antigens or infectious particles.

The factors explaining the induction of neurological disease in a single specimen are uncertain. A particular viral–host interaction clearly influenced the course and outcome of the infection in this individual, as in a similar manner with the rare natural cases of USUV clinical disease with encephalitis in humans [8,37]. Larger group sizes would be needed in future experiments to express the morbidity and mortality rates in relevant percentages. Specific mutations in USU-BE-Seraing/2017 [4] involved in an increased neuroinvasiveness and/or neurovirulence cannot be ruled out. The experimental infection of 129/Sv mice using this strain as well as the prototype strain SAAR-1776, which showed potential virulence in wild-type CFW mice [2], would shed light on the genetic determinism of USUV pathogenicity in this model. I.d. inoculation could have also been implicated in the outcome of the infection, as initial virus dissemination differs according to the injection route. Moreover, although we used a higher viral dose compared to that used by Martín-Acebes et al. [23], no signs or mortalities were observed following the i.p infection. In fact, initial replication of arthropod-borne flaviviruses is thought to occur in skin Langerhans dendritic cells following a mosquito bite or a needle inoculation via the cutaneous route [26,29,38]. The infected Langerhans cells migrate from the epidermis to the local draining lymph nodes [39] resulting in primary viremia and initiating the immune response [29,40]. TLR7 innate signaling in mouse keratinocytes not only plays a role in the host defense but also in WNV pathogenesis by promoting Langerhans dendritic cell dissemination from the skin to other peripheral organs [41], whereas it contributes to reduced viremia and lethality when WNV infection of mice is initiated by i.p. injection [41]. Natural infection is more complex than an intradermal injection, due to concurrent injection of the virus intravascularly [42] and of components of mosquito saliva [43,44,45] by the mosquito while probing and feeding on a live host. The effect of natural USUV infection in murine models needs to be explored.

No evidence of virus circulation was found in the blood in mice by RT-qPCR 15 days following the infection. However, the USUV genome was detected in their brains, in contrast to the study of Blázquez et al. [21], in which no USUV RNA (SAAR-1776 strain) could be detected from 8-week-old Swiss mice at any tested time after infection (4 to 35 days). Primary means of USUV entry to the brain are still to be determined. The pattern of WNV spread into the CNS may include both hematogenous or neuronal routes [46] and vary according to the route of inoculation [47]. In Vero cells, USUV can establish a persistent infection for at least 80 days [48]. USUV persistence in the brain and other organs of mice should be assessed by more prolonged experiments as well as possible delayed-onset disease.

The finding that in 129/Sv mice, the i.n. infection is able to spread the virus to other body compartments, especially the brain, is unprecedented and raises the possibility of close contact transmission of USUV in humans. This hypothesis is reinforced by the results of Vielle et al. [49], which showed that human respiratory epithelial cells of the nasal cavity are targets for USUV replication in vitro. Intranasal infection of immunocompetent mice with certain WNV strains resulted in fatal encephalitis and death of the animal [47,50,51,52], and in avian models, bird to bird transmission of WNV was experimentally confirmed [53]. A histopathological study including sections from the nasal cavity epithelium and the CNS (notably the olfactory bulb) at different stages of the infection would be needed to discern lesional patterns compatible with USUV replication in vivo. Further, virus shedding from the upper respiratory tract and contact transmission of USUV should be explored using this murine model. The i.n. route resulted in higher RNA loads in the brain compared to the i.p. and i.d. routes, which could be explained by the direct axonal transport of USUV from the olfactory neurons, as described for WNV [54].

Virus shedding via urine and feces could not be detected either by RT-qPCR nor cell culture at any stage of the infection. This indicates that the fecal-oral transmission of USUV is unlikely to happen in this model in our experimental conditions.

While all mice were negative for antibodies against USUV at the beginning of the experiment, positive or doubtful reactions were observed in the majority of the mice. This is indicative of viral replication and in accordance with the viral RNA being detected in the brains of all mice. The number of seroconverting specimens using the i.n. route was relatively higher compared to that in the intraperitoneally and intradermally infected groups. In general, the i.n immunization route favors the induction of strong immune responses with vaccine candidates against some important flaviviruses in human medicine [55,56]. The relatively high inoculation volume likely resulted in some of the virus dripping into the oropharynx and lungs, which could have also contributed to the enhanced dissemination of the virus and antibody response induced by this route. The high RNA loads maintained in the brain of intranasally infected mice 15 days following the infection in spite of the serological immune response can be explained by the function of the blood-brain barrier (BBB). Indeed, the BBB represents a highly selective interface between the circulating blood and the brain parenchyma and restricts the movement of substances, including antibodies, from the systemic circulation to the brain [57,58]. While animals injected via the i.p. route did not show particularly higher seroconversion rates 15 days post-infection, they had limited viral loads in their brains compared to the others, which is likely linked to a lower rate of viral replication in these individuals rather than an efficient viral clearance.

## 5. Conclusions

To our best knowledge, this is the first report of USUV experimental infection in mice using the i.d and i.n. routes. Overall, the 129/Sv mouse model showed a variable susceptibility according to the route of injection of USUV. Almost all mice survived to the experimental challenge with USUV but developed a neuroinvasive infection and a detectable antibody response. The i.d. injection of USUV strain USU-BE-Seraing/2017 caused severe neurological disease in a single mouse. The i.n. route turned out to be most efficient in terms of antibody-response induction and viral persistence in the brain of mice infected with both USUV strains but failed to elicit a clinical disease in our conditions. This pilot study gives grounds for further investigations regarding USUV direct transmission and the spatiotemporal process of neuroinvasion and neurovirulence of USUV strains using the i.d. and i.n. routes.

## Figures and Tables

**Table 1:** Antibody response against USUV infection tested by competitive ELISA in experimentally infected mice.

	USUV Strain					
	USU-BE-Seraing/2017			USU-BE-Grivegnee/2017		
**Infection route**	**P**	**N**	**D**	**P**	**N**	**D**
Intraperitoneal	2	1	3	1	1	3
Intradermic	2	0	4	3	1	2
Intranasal	5	0	1	4	0	2

D: doubtful; N: negative; P: positive.
